# A qualitative exploration of patient and clinician needs and preferences for a physical activity intervention during breast cancer chemotherapy

**DOI:** 10.1186/s12885-025-15342-y

**Published:** 2025-12-12

**Authors:** D. Carolina Andrade, Loni Parrish, Courtney Harriss, Lindsay L. Peterson, Ryan P. Duncan, Jingqin Luo, Maura M. Kepper, Christine Marx, Mary C. Politi, Elizabeth A. Salerno

**Affiliations:** 1 Division of Public Health Sciences, Department of Surgery, Washington University School of Medicine in St. Louis, St. Louis, MO 63110 USA; 2 Department of Population Health, University of Kansas Medical Center, Kansas City, KS 66103 USA; 3 Division of Medical Oncology, Department of Medicine, Washington University School of Medicine in St. Louis, St. Louis, MO 63110 USA; 4 Alvin J. Siteman Cancer Center, Washington University School of Medicine in St. Louis, St. Louis, MO 63110 USA; 5 Program in Physical Therapy, Washington University School of Medicine in St. Louis, St. Louis, MO 63110 USA; 6 School of Public Health, Washington University in St. Louis, St. Louis, MO 63130 USA; 7 Program of Applied Exercise Science, School of Kinesiology, University of Michigan, Ann Arbor, MI 48109 USA

**Keywords:** Physical activity, Chemotherapy, Cancer survivorship, Prehabilitation

## Abstract

**Background:**

Early intervention with physical activity (PA) during active cancer treatment is critical for improved health outcomes, but often difficult to deliver. We assessed patients’ and clinicians’ needs and preferences for a PA intervention during chemotherapy via semi-structured interviews.

**Methods:**

We conducted virtual semi-structured interviews from May 2022 to May 2023 with patients with stage I-III breast cancer and oncology clinicians recruited from a Midwestern comprehensive cancer center and community settings. We identified themes using deductive and inductive thematic analysis informed by the Consolidated Framework for Implementation Research.

**Results:**

Patients (*n* = 16) were mostly White, non-Hispanic (93.8%) females (100%) with an average age of 56.0 ± 12.0 years. Clinicians (*n* = 11) were primarily medical oncologists (45%) with > 10 years of experience (54%). Participants overwhelmingly viewed PA programs as beneficial and stated that they should be integrated into standard care during chemotherapy. Participants felt that if PA programs were presented as part of treatment, patients would be more likely to engage and adhere. Regarding intervention structure, patients preferred a flexible program that allowed them to decide when and how they would complete PA. They desired remote activities to increase the convenience of the program and overcome physical and logistical barriers such as chemotherapy side effects and transportation. They also wanted some in-person group elements for social support and accountability, with individual options for patients concerned with potential disease exposure. Clinicians expressed strong support for PA programs delivered by a physical therapist with oncology training and automated referrals in the electronic health record.

**Conclusions:**

Both patients and clinicians support PA programs delivered during chemotherapy. Key features of desirable PA programs were supervision by physical therapists, flexibility and convenience with both in-person and remote options, and group and individual components to facilitate patient participation. These findings can inform the design and implementation of future PA programs during chemotherapy for breast cancer.

**Supplementary Information:**

The online version contains supplementary material available at 10.1186/s12885-025-15342-y.

## Background

Breast cancer is the second-most common cancer in women in the United States, accounting for 30% of all new female cancers each year [1]. Patients receiving local and/or systemic treatments for breast cancer have reported numerous physical and emotional side effects, including depression and declines in mental acuity [2, 3]. Physical activity (PA) could be an important intervention to improve physical and mental health during and after treatment [4–7]. Physically active breast cancer survivors report fewer depressive symptoms than physically inactive survivors [7], and PA is associated with a reduced risk of cancer recurrence and mortality [4]. There is also emerging evidence of the benefits of PA on cognitive function in cancer survivors [8, 9]. 

Early intervention (prehabilitation; i.e., implementing PA before or even during chemotherapy) can help avoid complications and reduce treatment side effects including cognitive impairment [9]. However, the time between diagnosis and treatment initiation is complex, marked by significant time demands, frequent clinical appointments, and emotional overwhelm, which in a previous study was a significant barrier to program implementation [10]. Recruitment efforts for PA interventions during this period are also hampered by organizational barriers including lack of referral infrastructure and low buy-in from clinical teams [4, 11], as well as the perception that exercise for cancer patients is an “auxiliary” issue [11]. There is a need to clarify the relationship between mental health, cognitive function, and PA and learn potential participant preferences to know how best to intervene and achieve successful implementation during this complex time.

As part of a larger observational study examining the relationship between PA and cognitive and physical health during breast cancer chemotherapy and surgery (IMPACT Breast Cancer, NCT06595901), this study explored patients’ and clinicians’ perceptions of prehabilitation PA programs. Understanding their preferences and needs for the design (e.g., delivery mode, timing) and implementation (e.g., interventionists, clinic workflow) of a healthcare-integrated prehabilitation PA intervention is critical to inform and scale a behavioral intervention in the perioperative breast cancer setting that is acceptable, efficacious, and sustainable.

## Methods

We conducted semi-structured interviews from May 2022 to May 2023 with patients with stage I-III breast cancer and clinicians with oncology experience recruited from a Midwestern comprehensive cancer center and surrounding community settings. All materials and procedures were approved by the Institutional Review Board (IRB) at Washington University in St. Louis (IRB #202201163), and verbal consent was obtained prior to all interviews.

### Recruitment

*Patients with breast cancer*: Patients were approached during clinic visits by collaborating physicians and clinical coordinators to assess interest in the parent IMPACT Breast Cancer study. Interested patients then either contacted the study team directly or allowed clinic staff to share their contact information with the study team. Once contact was established, the study coordinator described study activities, shared a copy of the informed consent document via mail or email, and verified eligibility (confirmed diagnosis of breast cancer (stage I-III); female over the age of 18; scheduled to receive curative-intent breast surgery and chemotherapy (neoadjuvant or adjuvant); and able to understand and willing to agree to an IRB-approved waiver of documentation of consent). Detailed methods have been published previously [12]. Briefly, study activities for all enrolled patients (*n* = 30) included: using a hip-worn accelerometer for the duration of their chemotherapy and completing short questionnaires about cognitive function and mood throughout the duration of their chemotherapy. As this study was observational in nature, no additional PA prescription or programming was given. At the time of enrollment, the study coordinator invited all patients to participate in individual virtual (phone or videoconference) semi-structured interviews at any time during the study period. Patients were told that the purpose of the interviews was to better understand patient needs and preferences for future PA programs during chemotherapy. Patients who interviewed were compensated with an additional $20 gift card.

*Clinicians*: The study team shared promotional flyers and brochures with clinicians (i.e., oncologists, physical therapist, nurse coordinators) within the department of medical oncology and clinical community groups. Interested clinicians contacted the study coordinator by phone or email. The coordinator then explained the project, verified eligibility (i.e., being a healthcare professional with interest in improving quality of care during breast cancer treatment and able to understand and willing to agree an IRB-approved waiver of informed consent document), and scheduled the interview at a time of the participant’s choosing. Clinicians who interviewed were offered a $20 gift card.

### Procedure

Individual semi-structured interviews with patients and clinicians were conducted by phone or video conferencing by mostly female study team members, including the study coordinator (CH), principal investigator (ES), a postdoctoral student (LP), and undergraduate research assistants, all of whom were trained and experienced in semi-structured qualitative interviewing procedures. Interviews were capped at 45 min to reduce participant burden. Interview guides were informed by the Consolidated Framework for Implementation Research (CFIR) [13] and adapted for this population through feedback from a multidisciplinary advisory board. After establishing rapport, the interviewer asked questions addressing all five CFIR domains (i.e., intervention characteristics, inner and outer setting, characteristics of the individuals involved, and process for implementation). Clinicians were asked additional questions about the inner setting (e.g., direct and indirect costs of implementation, organizational culture and readiness—see Supplementary material 1 for full guide). Interviews were recorded and transcribed for thematic analysis.

### Analysis

The study sample size (*n* = 30) was selected to satisfy both the need for theme saturation and pragmaticism due to chemotherapy timing constraints [14]. Transcripts were analyzed thematically using deductive and inductive methods. First, the study team (DCA & ES) created an initial codebook of a priori codes based on the interview guides and CFIR domains. More specifically, the CFIR domains were master/“grandparent” codes; each question pertaining to a CFIR domain was made a parent code; and key, repeated ideas from participants were sub-codes. For example, the process domain was a master code with four parent codes: “facilitate referrals,” “recruitment/enrollment tips,” “reasonable,” and “other considerations”, with some containing additional children. Next, the preliminary codebook was tested during initial coding of four transcripts (two from each interview type) and refined inductively as study team members reviewed and coded all remaining transcripts in NVivo 14. Coders (DCA & LP) met bi-weekly to discuss coding discrepancies, refine coding structure (particularly sub-codes), and reach consensus. After all transcripts were coded, all codes were grouped into categories and discussed to identify the most salient themes. Finally, participants’ demographic characteristics were analyzed in R (R version 4.4.1).

## Results

Of the 30 patients and 12 physicians approached, a total of 28 participants interviewed: 17 patients and 11 clinicians. However, one patient interview was unable to be analyzed due to a problem with transcription, for a final patient sample of 16. Most patients (Table 1) identified as non-Hispanic (93.8%) White (87.5%) and were on average 56.0 ± 12.0 years of age. Clinicians (Table 2) had a range of expertise; most were medical oncologists (45.5%) who had been in practice for more than 10 years (54.5%).

**Table 1:** Patient demographics

	Overall (*N* = 16)
Age (years)	
Mean (SD)	56.0 (12.0)
Median [Min, Max]	57.5 [37.0, 73.0]
Race	
Black or African American	2 (12.4%)
White	14 (87.6%)
Ethnicity	
Hispanic or Latino	1 (6.2%)
Non-Hispanic or Latino	15 (93.8%)

**Table 2:** Clinician demographics

	Overall (*N* = 11)
Role	
Medical Oncologist	5 (45.5%)
Advanced Practice Provider	2 (18.2%)
Psychiatrist	1 (9.1%)
Nurse	1 (9.1%)
Registered Dietician	1 (9.1%)
Physical Therapist	1 (9.1%)
Experience	
5–10 years	5 (45.5%)
>10 years	6 (54.5%)

Four key themes emerged from the semi-structured interviews (Table 3).

**Table 3:** Key themes and illustrative quotes from IMPACT semi-structured interviews (*n* = 27)

Theme	CFIR Domain/Construct^13^	Relevant Codes	Illustrative Quotes
1. A PA program during chemotherapy is desirable and beneficial and should be offered as part of standard care	Characteristics of Individuals: Knowledge and beliefs about the intervention Inner setting: Implementation climate	PA programs during cancer treatment - desirable	“I feel very strongly that that [PA] should absolutely be a part of the treatment for every patient at the time of diagnosis.” – *Physician Assistant* “You package it as part of the treatment plan. Just another aspect. Just like chemo. Don’t say, ‘Hey, this is an extra resource that we offer.’ Because I’m here to tell you, that binder they handed me with all the extra resources, I scanned it, it went in a drawer, and I haven’t looked at it since… If you want something to stick, attach it to the treatment plan.” *- Patient (Stage I)* “I think it should be offered as a standard component… this should be incorporated into our treatment plan... because I think [it] just does help with several different aspects of patients' health.” *– Oncologist* “I think it needs to be prescribed as part of the treatment plan... if you had intervention at the very beginning before people start chemotherapy, and it’s pitched as part of the whole treatment plan, and they know going in their ability to tolerate the chemicals will improve if they engage in moderate exercise or activity.” *– Patient (Stage III)* “Oh, I think that would be very good… an integral part actually, not just complimentary… this notion of physical activity… is holistic. I think that’s very important, it’s not just for the body, it’s for the mind and it comes at a very important time.” *– Patient (Stage I)* “As soon as they know that they have cancer, that should be one of the first things that you would bring up right there, in the doctor’s office, when you’re going through, ‘Well, this is the treatment that we would like for you to do. These are the suggestions.’” – *Patient (Stage I)* “I think it’s definitely needed, because now it’s almost like I’m trying to relearn how to have stamina to do the tasks that I felt were normal.” *– Patient (Stage I)*
		PA programs during cancer treatment - beneficial	“I think it’s the most valuable part ever. I don’t think I would be doing as well if I wasn’t pushing myself to make sure that I was being active.” *– Patient (Stage I)* “I think it’s allowed me to tolerate and continue to tolerate the chemotherapy.” *– Patient (Stage III)* “I know once I just got up and kept moving, you get a little bit more energy. Even in the thick of that chemo, just keep on moving and moving. I felt like I had better energy.” *– Patient (Stage II)* “I think it would be good for physical activity to be part of the breast cancer regimen, because—there are obviously definite days that you don’t feel like doing things, but I think sometimes those are the days that you probably need to do them the most, is to get up and get moving and try to help your body move along.” *– Patient (Stage II)* “I think it’s very important. Again, we have the support for the mental, the physical, the nutrition, but I think that that[exercise] is one thing that is lacking, and I think it has such an impact on your emotional and mental health as well as, obviously, your physical. I think that it would be a huge service to patients to be able to offer them that kind of intervention.” – *Dietician* “I think it would meet the needs of patients very well. I really do and I think they’d be very receptive to it. I think they would see a lot of benefit from it to their physical health and their mental health.” – *Nurse Practitioner* “I would think that they would want to do it, and I think it would be good for them. I think it would be good for anybody on—like I was saying before, I think the worst thing somebody can do is just lay around and do nothing.” – *Patient (Stage II)*
2. Clinician endorsement and delivery professionals with oncology experience would increase program buy-in	Process: engaging Intervention characteristics: Design quality & packaging	Recruitment - clinician	“The number one thing that would get me in a program would really be the recommendation of my healthcare providers. If my healthcare provider said, ‘Look, there’s this program… this would benefit you. I think you would get positive results from this’… I would’ve tried to find a way to do it.” – *Patient (Stage I)* “For sure, the doctor has to encourage you to participate. The doctor has to tell you, ‘I’m the person overseeing your treatment, and I think this would help you.’” – *Patient (Stage I)* “What would make me do it? Somebody telling me to, the benefits statements associated, showing where I’d be with and where I’d be without, that can be pretty powerful.” – *Patient (Stage I)* “The healthcare provider can tell you… I listen to everything they said because I wanna live on the other side of this… Once… they tell you to do something, people have to adhere to that. They really do.” – *Patient (Stage II)* “When I first start and everybody’s telling me, that makes me more interested. Like, the doctor might say something, and then you[program staff] can come in and follow up and tell me more, so that’ll make people interested even more.” – *Patient (Stage I)* “Clearance from their primary or preferably their oncologist if they're in chemotherapy... that they're able to participate in a resistance and/or aerobic training regimen... I think that would be really helpful.” – *Physician Assistant* “What do I think helps to get people to enroll in it? Again, just lots of information, lots of statistics, stories, just saying how beneficial it is.” – *Patient (Stage II)*
		Delivery professional	“… a physical therapist or physical trainer, somebody who has the experience and the knowledge for the exercises, somebody who is familiar with working with breast cancer patients that would know what some of their limitations could be…” – *Patient (Stage I)* “… if you have somebody who physical activity or exercise is their expertise, then they will know how to… make it appropriate for people at all different abilities, all different stages during chemo, all different stages of physical fitness when they start chemo, all of that.” – *Dietician*
3. Chemotherapy-related side effects, logistics, and cultural beliefs are significant participation and implementation barriers but social support a key facilitator	Outer Setting: patient needs and resources Inner Setting: Culture	Barriers - chemotherapy side effects	“For me, the biggest thing about the side effects have been the fatigue. Weakness, drained, fatigue have been the absolute worst for me… Just the exhaustion... It’s been probably three weeks since I worked out last because I just don’t physically have the energy—even mental energy—to force myself.” – *Patient (Stage I)* “Some people even have difficulty walking… Some people go home with a pump. I don’t know that they could do this. Could I ride my bike if I was hooked to a pump?… how well can you ride your bike, if you’re really throwing up all the time? I know it’d be really hard for some people.” – *Patient (Stage I)* “… not feeling well. Is it your GI giving you problems or just maybe sometimes being down in the dumps, cutting through that? How would I handle crossing that threshold and doing it anyway?” – *Patient (Stage I)* “I think your barrier’s gonna be people are gonna be very tired. That’s all I can tell you from chemo is that I’m very tired and that it’s very hard to add in something like that.” – *Patient (Stage II)* “… the way it attacks your body feels and the fatigue—if you had something regimented—there’s days I don’t think I could’ve done any of that.” – *Patient (Stage II)*
		Barriers - logistical	“Transportation comes to mind… I know there are people that don’t have their own vehicles or can’t drive for themselves, rely on friends or whatever. That might be hard getting for them.” – *Patient (Stage I)* “I live an hour from the closest… center. For treatment, to drive there once every three weeks isn’t an issue, but to drive there what I’m assuming is at least a few times a week for physical activity is not really doable in my life to work all day and have a family. I could see that being a barrier.” – *Patient (Stage II)* “I’d say the time commitment with all of those appointments, especially if we’re trying to get somebody in before surgery…” – *Physical Therapist* “Sometimes some of the providers can only do it midday, and that doesn’t work for people that are working.” – *Dietician* “... if there’s a big cost to it. That would discourage people.” – *Patient (Stage III)*
		Barriers - psychosocial	“There’s a whole lot that comes with breast cancer that is outside of just your treatment scope. It’s the mental weight. It’s the keeping up with paperwork and financial aspects on top of not always feeling fabulous. The guilt to also try to fit that [PA] in, I could see that being a downside.” – *Patient (Stage II)* “Family not wanting them to do everything, worried about that they’re gonna overdo… the biggest barrier is people, the families not wanting them to… people don’t think that you can do it, and they just, ‘No, no. Don’t do that…’” – *Patient (Stage II)*
		Barriers - institutional	“There’s a lot of apprehension about incorporating physical activity in a population that would seem to be unwell or… undergoing chemotherapy. Generally, there’s… pushback from that angle. From the healthcare system standpoint, the codes that are tied to reimbursement have to align with physical activity… and they often do not, unfortunately. There’s, I guess, less support from the culture in the healthcare [organization] for healthcare readiness aspect of it.” – *Oncologist* “Patients still feel that the overwhelming sense is like, ‘Oh, you’re getting chemo. You need to just rest.’… I think that’s starting to change, the institutional mindset, but yeah, it feels like historically… it wasn’t valued… wasn’t even thought of as a possibility.” – *Oncologist*
		Facilitators - social support	“I think having a buddy is always nice. I have learned... just through treatment you need a strong support system. Having someone that’s willing to support you in that, remind you that you can do it, they can be with you, I think that would be great.” – *Patient (Stage II)* “Having something that’s like a small group—two people or a small group—of people going through what you’re going through to help encourage each other along…” – *Patient (Stage I)* “If you could get… a program that everyone’s alike as far as we’re all going through the same thing because sometimes, you get your best encouragement with people that really do understand what you’re going through, or somebody that’s been through it. They get it… it’s better to encourage doing that.” – *Patient (Stage II)* “… friends or just people that—or a coach or somebody that checks in with you to say, ‘All right, even if you can’t make it to the gym, let’s just try a walk.’ That kind of encouragement, I think, can help get people over the hump.” – *Patient (Stage III)* “I think for exercising, particularly if you don’t have that as a habit, then it needs to have a social aspect to it.” – *Patient (Stage III)* “Definitely a support group and an accountability partner… somebody to make me get out and do it, but then still understand that there’s gonna be days that I don’t feel like it, I just don’t have it in me to do it.” – *Patient (Stage I)*
4. Participants desire accountability, flexibility, and personalization in intervention components, structure, and delivery mode	Intervention characteristics	Intervention components	“I think actually having the activity monitor on me, reminded me to be active…Literally having something physically around my waist that measured my activity level was a constant reminder to get up and try to be active, even if I didn’t feel like I had it in me.” – *Patient (Stage I)* “Having to track what I’m doing is motivating to me to do something. I think having a system where you have to be responsible for turning in information about your health, like what your activity levels are, tracking for the doctor or something like that, I think would be good.” – *Patient (Stage II)* “Low impact activity, walking for a set time or distance. Something that’s not overly time consuming, and even something like yoga or something like that for half an hour or so I think is manageable.” – *Patient (Stage II)* “One important piece would be behavior change counseling. I think that’s something that—it’s typically kind of the aspect of behavior change that are the hardest for people to make, and so I think the cognitive piece could help those people.” – *Physician Assistant* “ ... it could even be something on video, like YouTube. Somebody who’s maybe going through a YouTube exercise, or showing you how you could move without hurting yourself, or what’s a good move with having a new port.” – *Patient (Stage II)*
		Intervention structure - flexible/tailored to patient	“Making it more convenient for patients, tailor to patients. For example, their schedule being flexible, whether it’s onsite or remote. I think that would be helpful. […] I feel like making it more flexible, wherever they can do it that it doesn’t require a lot of instruments—or equipment—would be helpful.” – *Oncologist* “Trying to meet the patient where they are in terms of their ability to participate would be the key factor in determining where, what, when, and how.” – *Psychiatrist* “I think it should be a multipronged approach. That way the patients have the ability to pick and choose what fits their lifestyle.” – *Oncologist* “Flexibility in offering, and depending on the day because symptoms, it’ll vary. It’ll definitely vary how you feel day-to-day and what you’re up for. One day, we… did an extremely rugged two-mile hike. Then other days, it’s like I’m not crawling out of bed.” – *Patient (Stage I)* “I think it has to be very person-dependent… prescribed for the person that you have in front of you. Not everybody’s the same. Not everybody starts from the same point… I think that patients need to be… evaluated for their physical status and then start prescribing a program that they can… maintain or modify depending on the energy.” – *Oncologist* “I think groups are wonderful—but always taking into account that you’re not prescribing the same physical activity program to all the patients because that cookie-cutter system doesn’t work.” – *Oncologist*
		Delivery mode	“I think giving them the option to either come in person or have a telehealth is a huge thing for most patients because they want that option to be able to do either one… Some people really want that in-person interaction as well, so I think it’s important to have both of those things.” – *Nurse* “On days that maybe I just don’t feel like getting out, for whatever reason, or if I got the sniffles, or whatever, I wanna stay away from people, then I could join via Zoom. I think a combination of the two would be really beneficial. That way people have the option.” – *Patient (Stage I)* “I like the idea of both. Like one-on-one with a personal trainer or a physical therapist sounds awesome. I like the idea of… a group or small group of walking buddies, or a small dance class…” – *Patient (Stage I)* “I would do individual, but it would be fun also to have a group, especially if it was other breast cancer participants…” – *Patient (Stage I)* “I’d prefer one-on-one. I think that would be important too for someone who may not be very physically active. Even people going to a gym, sometimes, it’s intimidat[ing] because they may not be in shape. I think one-on-one would be better.” – *Patient (Stage II)* “Group programs provide the benefit that the patients have the opportunity to bond with each other… they will even get together to do the physical activity together or encourage each other via text, things like that.” – *Oncologist* “If I did my chemotherapy and they said, ‘Okay, now before you leave [the treatment center], stop in here. We have a half hour yoga session, or we want you to do some time on the bicycle,’ I would do it in a second—if it was right there.” – *Patient (Stage I)* “I would think the community center or even if the—the treatment center… had a room available where they could have things, classes or whatever, that you could go and join, that would be good. In nice weather, there’s always the big parking lots that we can walk. That would be a good place to have it because we’re already used to going there.” – *Patient (Stage I)*

### Theme 1: A PA program during chemotherapy is desirable and beneficial and should be offered as part of standard care

#### PA is desirable and beneficial

Participants had an overwhelmingly positive response to the idea of delivering/participating in a PA program during chemotherapy. Nearly all patients and clinicians expressed the idea that being physically active during treatment is both necessary and beneficial, with several describing it as *“essential.”* Patients stated that this kind of intervention would *“improve or at the very least*,* maintain”* their physical condition, which, for most, worsened significantly during chemotherapy. As one patient explained,

*“I think it’s definitely needed*,* because now it’s almost like I’m trying to relearn how to have stamina to do the tasks that I felt were normal.” – Patient (Stage I).*

Patients felt that being physically active can and has improved their physical condition and their response to treatment, as these patients describe:

*“I think it’s the most valuable part ever. I don’t think I would be doing as well if I wasn’t pushing myself to make sure that I was being active.” – Patient (Stage III)*

*“I think it’s allowed me to tolerate and continue to tolerate the chemotherapy.” – Patient (Stage III)*

*“I know once I just got up and kept moving*,* you get a little bit more energy. Even in the thick of that chemo.” – Patient (Stage II)*

Patients noted that being physically active can also improve one’s mood during chemotherapy. Patients explained that in addition to increasing their confidence and decreasing their depression, being physically active *“makes you feel normal even though you’ve received this diagnosis.”*

Clinicians echoed patients’ views about the need for exercise during chemotherapy treatment and its physical and mental health benefits. Clinicians mentioned that *“a lot [of patients] are pretty sedentary… it just gets even worse during chemotherapy… there’s that big need for it.”* They mentioned that patients *“are always looking for it [PA programs]”* to contribute to their cancer treatment and healing. As one clinician explained,

*“Patients seem to really enjoy these types of intervention*,* because it gives them something that they could do to help potentially improve their outcome.” – Oncologist*

#### Standard part of treatment

Many patients stated that PA programs should be presented as a default part of the treatment plan—as “*[the] next therapeutic task.”* One patient elaborated on the importance of integrating the intervention into the treatment plan:

*“You package it as part of the treatment plan. Just another aspect. Just like chemo. Don’t say*,* ‘Hey*,* this is an extra resource that we offer.’ Because I’m here to tell you*,* that binder they handed me with all the extra resources*,* I scanned it*,* it went in a drawer*,* and I haven’t looked at it since… If you want something to stick*,* attach it to the treatment plan.” – Patient (Stage II)*

Though most clinicians agreed that PA *“should absolutely be a part of the treatment for every patient at the time of diagnosis*,*”* some spoke of the importance of presenting the program as optional and allowing patients to decide to participate. Clinicians recognized that patients may already feel like they have had little agency over the other aspects of their treatment like chemotherapy or surgery and may dislike further obligations. As one explained:

*“I’ve found that when people are… trying to be enrolled into… a lifestyle change program*,* but they’re not at all willing or ready to do that*,* it is not at all successful*,* and it actually can alienate them a little bit*,* and it can be a frustrating experience. I think the first thing is to advise patients of the need and purpose of physical activity*,* but then to follow up that question with*,* ‘Are you ready to work on this?’” – Physician Assistant*

### Theme 2. Clinician endorsement and delivery professionals with oncology experience would increase program buy-in

#### Clinician referral

More than half of patients stated that their clinician’s recommendation or referral was/would be the most persuasive factor during recruitment. Patients stated that their clinician’s endorsement of the program highlighted the importance of PA during treatment and assuaged safety concerns, motivating them to participate. As one patient explained:

“*I listen to everything they [clinician] said because I wanna live on the other side of this... Once... they tell you to do something, people have to adhere to that. They really do.” – Patient (Stage II)*

Most patients noted that they preferred to hear about the intervention from their oncologist or nurse during a clinic visit at the time of diagnosis or before starting chemotherapy. Participants were open to remote recruitment (i.e., over the phone or Zoom) with program staff, but most preferred to be informed of the study by their doctor’s office first.

Clinicians stated that time constraints and information overload were key challenges to recruitment during clinic visits during treatment initiation. To facilitate referrals, almost all clinicians suggested a direct referral system in the EMR, which one community-based nurse stated, *“tripled our referrals because they [doctors] had an easy way to put it in the system and get it to us.”* Clinicians and patients emphasized the need to make it as easy as possible for patients to enroll, meaning that the program staff would need to lead the outreach, management, and follow-up with both clinical teams and patients. Clinicians requested that program staff flag eligible participants and reach out to clinicians with patient names instead of waiting for providers to screen their clinics. They also stated that having program brochures and a dedicated contact person on the program staff to manage patient referrals would also assist recruitment efforts.

#### Delivery professional

Participants most often stated that they preferred a physical therapist to deliver this kind of intervention. Both clinicians and patients emphasized the need for the delivery professional to be experienced in oncology and adept at modifying exercise recommendations in response to physical limitations. As one clinician explained,

“… *if you have somebody who physical activity or exercise is their expertise, then they will know how to… make it appropriate for people at all different abilities, all different stages during chemo, all different stages of physical fitness when they start chemo, all of that.” – Dietician*

A few patients emphasized the importance of involving their clinical team in program supervision, as well as being able to relate to the exercise professional delivering the program. One patient stated:

“*You need… somebody that understands where we’re coming from… it’s older women that have breast cancer too that need to… be active too. We may be more limited than these younger people [who] have more energy.” – Patient (Stage II)*

### Theme 3. Chemotherapy-related side effects, logistics, and cultural beliefs are significant participation and implementation barriers but social support a key facilitator

The most common participation barrier identified by patients was feeling unwell because of chemotherapy. Extreme fatigue and medication side effects were identified as significant barriers to physical activity. Patients mentioned that tailoring program activities to individual patients’ strengths and physical condition would facilitate participation (*see Theme 4*).

Some patients also spoke of the mental toll of chemotherapy and how, combined, *“not feeling well”* and *“being down in the dumps”* did not motivate patients to participate. Several patients mentioned that feeling emotionally overwhelmed, embarrassed by their physical condition, or pessimistic about their diagnosis would also discourage participation. Patients emphasized the need for strong social support either in the form of peers with whom to exercise, dedicated coaches, and/or family buy-in to improve and maintain motivation, particularly when feeling low or unwell. A few patients mentioned that family can sometimes be a barrier to PA, as they discourage patients from exerting themselves, wanting them only to rest. Participants expressed interest in bolstering family support by educating family members on the benefits of PA for chemotherapy patients and involving them in the exercise program as well. As one participant explained:

*“If it was a program that your kids could be involved in too*,* that you could bring your kids and do an activity together… that’s another resource that might help…’”* – *Patient (Stage III)*

While patients focused on whether they would feel emotionally and physically well enough to participate, clinicians spoke more about logistical and cultural barriers (i.e., time, staffing, healthcare system views, and cost). Clinicians mentioned that *“some patients… probably can’t make it work because they just don’t have any extra time”* between their home, work, and treatment obligations. Transportation and distance to the program locale were also identified as key barriers to participation. The logistical supports suggested included rideshares, stipends for travel expenses, and/or remote delivery options.

Clinicians also cited organizational barriers to implementation, such as staffing shortages, current clinical habits, and negative or ambivalent cultural views on the value of PA programs and on PA for chemotherapy patients in general. As these clinicians explained:

*“… the healthcare system probably doesn’t prioritize it*, *which is why it feels like there is always a worry that these [programs] aren’t gonna be reimbursed… I think that’s starting to change*, *the institutional mindset*, *but yeah*, *it feels like historically… it[PA] wasn’t valued… wasn’t even thought of as a possibility.”* – *Oncologist*

*“There’s a lot of apprehension about incorporating physical activity in a population that would seem to be unwell or… undergoing chemotherapy. Generally*, *there’s… pushback from that angle. From the healthcare system standpoint*, *the codes that are tied to reimbursement have to align with physical activity…* *and they often do not*, *unfortunately.”* – *Psychiatrist*

*“I think that assumptions about patients’ abilities*, *willingness*, *readiness… will definitely influence that[referrals]… we tend to assume that patients are not willing and able or wanting to do these types of interventions*, *so I think that could definitely kind of impact who is referred or recommended to participate in these programs…”* – *Physician Assistant*

Many clinicians expressed concern about referring patients to a program that may not be covered by insurance. To address this, clinicians spoke of the need to make the program cost-free or low-cost and to be transparent about potential out-of-pocket payments and the availability of financial assistance at their treatment center. Some clinicians mentioned that the best way to change the cultural value placed on PA during chemotherapy would be to provide empirical evidence of its benefits and allow clinicians to participate in the physical activity program as well:

*“Make it available to the physicians and the staff…if you say*, *‘Hey*, *you can try it out and… you can see the individualized benefit yourself.’ […] I think then you’ve kinda built a culture of the cancer center where not only the patients are seeing the benefit*,* the employees are seeing the benefit in it.”* – *Oncologist*

“… *with the data being so strong*, *as strong as it is*, *I think that a lot of specifically oncology clinicians would… look to this intervention very favorably…” – Physician Assistant*

### Theme 4. Participants desire accountability, flexibility, and personalization in intervention components, structure, and delivery mode

#### Components

Many patients expressed a desire to use activity trackers, activity journals, or online progress boards to monitor their progress and provide accountability throughout the intervention. As one patient explained,

*“I think actually having the activity monitor on me*,* reminded me to be active… Literally having something physically around my waist that measured my activity level was a constant reminder to get up and try to be active*,* even if I didn’t feel like I had it in me.” – Patient (Stage I)*

Patients also stated that receiving texts, emails, or push notifications served as helpful reminders to move and as recognition for their efforts, which encouraged them to continue. Other intervention materials requested included physical exercise equipment and online or printed educational resources demonstrating the correct technique for different exercises to ensure safety, explain potential health benefits derived from each exercise, and accommodate specific treatment-related needs:

*“… it could even be something on video*,* like YouTube… showing you how you could move without hurting yourself*,* or what’s a good move with having a new port.” – Patient (Stage III)*

Though walking was the most common activity suggested by patients, most felt that a range of home-based activities should be offered to meet individual interests and capabilities, including yoga, calisthenics, recumbent bike, dancing, and weight-bearing exercises. Patients also expressed a preference for short bouts of activity (~ 30 min) two or three times a week. 

#### Structure

The most common request was for the intervention to be flexible and customizable in both content and delivery. A few participants expressed interest in a regimented group program to bolster a sense of accountability and support. Others expressed concerns about how much they would be able to participate in a structured program due to their physical condition and instead emphasized that the program should consist of *“what the individual feels that they could do.” * They expressed a need for a range of activities or tailored instruction. Some patients felt that the program should be guided by individual goals and that these should decide the program’s frequency, activities, and intensity. Clinicians also noted the importance of tailoring the regimen to the individual’s goals, priorities, resources, and physical limitations to maximize success. As one clinician described,

*“I think it has to be very person-dependent… prescribed for the person that you have in front of you. Not everybody’s the same. Not everybody starts from the same point… I think that patients need to be…evaluated for their physical status and then start prescribing a program that they can… maintain or modify depending on the energy.” – Oncologist*

Notably, clinicians cautioned against framing PA programs as weight-loss interventions, which can compound feelings of guilt in cancer patients, as a nurse practitioner explained:

*“A lot of women… if they are somewhat overweight… feel bad. They feel like ‘okay*,* maybe if I were fit and healthy all of these years I wouldn’t have gotten breast cancer.’ They’re already blaming themselves… so it needs to be physical activity*,* yes*,* but in a very positive lens of health*,* not like*,* ‘You need to walk every day to lose weight.’” – Nurse practitioner*

#### Delivery Mode

Most patient participants preferred a small group-based program, citing benefits such as enhanced motivation, encouragement, enjoyment, fun, and improved accountability and adherence. Many mentioned that they would prefer the group to consist of only patients with cancer because “*it’s nice to talk to other people who are in the same situation as you.” * A few patients identified emotional barriers to group delivery, such as feeling *“awkward”*,* “self-conscious”*, or “*intimidated because they may not be in shape*.” An additional concern about group-based programs was that patients might not all be at the same stage of treatment or physical condition, which might affect others’ motivation levels:

*“If I’m with a group of people that’s [in] chemotherapy—they’re in a lot worse shape than I am… then I won’t be as motivated to work as hard.”* – *Patient (Stage II)*

Those who preferred individual delivery shared that it would be easier to coordinate, less intimidating, and had the potential to be more motivating, as patients felt like they *“had an appointment to keep.” * These participants expressed a preference for offering both individual and group activities and allowing patients to decide what they want to attend. One clinician emphasized that group-based programs, despite their synchronous delivery, should still “*not prescribe the same physical activity program to all the patients because that cookie-cutter system doesn’t work.*”

The group vs. individual delivery discussion was often conflated with the choice between in-person vs. remote delivery modes. Overall, participants indicated interest in having both options available to best accommodate diverse patient needs, comfort levels, safety, and resources. Several patients suggested that in-person physical activity could be offered at their treatment center:

“*If I did my chemotherapy and they said*,* ‘Okay*,* now before you leave*,* stop in here. We have a half hour yoga session*,* or we want you to do some time on the bicycle*,*’ I would do it in a second—if it was right there.”* – *Patient (Stage III)*

Notably, almost all participants expressed a high degree of interest in synchronous virtual programming (i.e., over Zoom videoconferencing) because of its increased convenience and flexibility, which would facilitate participation when experiencing adverse reactions to chemotherapy. Participants felt that virtual delivery would allow broader dissemination, reduce costs, improve attendance, and decrease exposure to diseases such as flu and COVID-19. Challenges to virtual delivery included motivation if asynchronous, perceived judgement from family members due to a lack of privacy at home, and low tech literacy or limited internet or computer access.

## Discussion

This study aimed to explore patients’ and clinicians’ perceptions of the best ways to design, deliver, and motivate patients with breast cancer to engage in a large-scale, healthcare-integrated PA program while actively undergoing chemotherapy. This information is critical to inform and scale a behavioral intervention in the perioperative breast cancer setting that is acceptable, efficacious, and sustainable. We found that patients and clinicians had high interest in PA programs during active treatment, and some believed they should be offered as part of standard cancer care. Most patients preferred group-based PA programs with flexible delivery formats (i.e., virtual and in-person) and tailored PA content. Patients and clinicians emphasized the need to tailor exercise plans to individual patient interests and abilities for best adherence and outcomes. Key supports desired included wearable activity trackers for accountability, counseling/a direct referral from their oncologist, and supervision by a physical therapist with oncology training. Finally, numerous logistical, individual, and organizational barriers to participation were identified.

The most desired attribute for PA programs during chemotherapy was flexibility in terms of remote (home-based) vs. in-person (community-based) program delivery, which was heavily conflated with group vs. individual delivery. Patients and clinicians appreciated the potential social benefits of group delivery (i.e., increased support, camaraderie, and accountability for patients); however, nearly all participants were equally interested in the logistical benefits of remote delivery, which they felt might help overcome chemotherapy-related participation barriers. While remote, home-based interventions may improve adherence and access by removing transportation and time barriers [15, 16], there is evidence that they are less effective than interactive, community-based group PA programs [8, 16–18]. Interactive programs allow for higher exercise doses to be delivered, as patients can receive more attention, motivation, reinforcement, and support in-person [8]. However, some patients do not wish to identify as cancer survivors and would prefer privacy and autonomy over PA during recovery [5]. Thus, our results support hybrid models, with supervised in-person and group activities at convenient locations such as treatment centers to provide patients with the social support and accountability they desire, but also allow for remote and individual participation to provide patients flexibility and autonomy.

Patients and clinicians also expressed a desire for individualized exercise regimens, even in the context of a group program. This aligns with current American College of Sports Medicine (ACSM) guidelines, which recommend that exercise professionals consider patients’ cancer type, stage, chemotherapy regimen, comorbidities, functional status, age, and goals to prepare exercise plans customized to patients’ needs [8]. This tailored approach would help abate patient concerns about being physically unable to complete program activities during active treatment [10]. Exercise professionals should consider utilizing exercise testing to gauge exercise tolerance [13]​ and discuss patients’ PA resources and interests to develop feasible, patient-driven exercise plans. The inclusion of health behavior theory in these plans can further support self-efficacy and self-regulation for long-term PA maintenance.

Chemotherapy-related side effects were identified as the most significant physical and emotional barriers to participation. Our participants reported that the belief that chemotherapy patients should avoid exertion, which was historically the clinical standard [5, 8, 10, 19], led well-meaning family members and clinicians to deprioritize exercise during treatment. It is critical to provide evidence of the safety and benefits of PA interventions to patients, caregivers, and clinicians to improve PA uptake and adherence [7, 8] during chemotherapy and erode prevailing beliefs to the contrary [19]. Given that patients generally regard physicians as authority figures and rely on their guidance [20], this power dynamic could be positively harnessed to encourage PA. Indeed, there have been numerous calls for physician engagement to assess, advise, and refer patients to the appropriate physical activity resources [19]. Our findings reinforce the need for additional care coordination, which would benefit from further research into the behavioral and implementation barriers to PA integration in cancer care.

As for institutional implementation barriers, our clinicians highlighted insurance reimbursement, the lack of a streamlined referral process, time constraints, and a culture that prioritizes therapeutics over lifestyle interventions as key barriers to recruitment and institutional support for PA programs during chemotherapy. Many of these cultural and logistical barriers to PA programs for cancer patients have been well-documented [4]. To minimize the insurance reimbursement concern, exercise specialists typically covered by most insurance policies (e.g., physical therapists; PTs) can be employed to deliver a PA intervention [21]. PTs have the additional benefit of engendering more trust in patients as a hospital-affiliated, oncology-referred service [22], and their demonstrated success with telehealth [23] may further improve PA uptake and adherence. To mitigate logistical and time barriers to recruitment in clinic, program staff (e.g., clinical research coordinators) must lead efforts to identify potentially eligible participants, so that physicians can simply reinforce PA referrals rather than introduce them during patient encounters. Integrating a standardized or automatic referral system into existing clinic workflows would also improve program implementation. To incentivize institutional support to build such program and recruitment infrastructure, studies exploring the cost-effectiveness of PA interventions during chemotherapy in U.S. contexts are needed.

## Limitations & strengths

This study was subject to selection bias, as only highly motivated patients self-selected and adhered to the parent study. Additionally, it is not known how physically active patients were prior to enrollment; women with an extensive history of PA behavior may have had more favorable views of integrating PA into cancer care and/or perceive there to be fewer barriers to accessing PA programming during active cancer treatment. Patients were interviewed at any point after initiating the intervention; while most were interviewed within a few weeks of initiation, some were interviewed while offboarding. The timing may have affected patient responses, as newcomers may have been more favorable than patients offboarding, or vice vera. The generalizability of results may be limited, as patients were only recruited from one academic hospital system in the Midwest, and the sample size was both small and lacked racial and ethnic diversity. Still, this study presents valuable insights for the development and implementation of future PA programs during chemotherapy for breast cancer. The inclusion of both patient and clinician perspectives is a particular strength of the study. Future studies should incorporate these preferences in a large, randomized pilot feasibility study and evaluate patient adherence.

## Conclusions

This qualitative study found that both patients and clinicians support PA programs delivered during chemotherapy for breast cancer. Key features of desirable PA programs were supervision by physical therapists, flexibility and convenience with a mix of in-person and remote options, and group and individual components to facilitate patient participation. Addressing institutional barriers through PA educational campaigns and streamlined referral processes may improve recruitment and physician buy-in. These findings can inform the design and implementation of future PA programs during chemotherapy for breast cancer.

## Supplementary Information

Supplementary Material 1.

## Data Availability

The datasets generated and/or analyzed during the current study are not publicly available due to patient confidentiality, but a codebook is available from the corresponding author on reasonable request.

## Abbreviations

PA: physical activity
