# Supplementary material for: A qualitative exploration of patient and clinician needs and preferences for a physical activity intervention during breast cancer chemotherapy
Source: BMC Cancer. 2025 Dec 12;26:238. doi: 10.1186/s12885-025-15342-y (PMC12903211; doi:10.1186/s12885-025-15342-y)
Supplement: Supplementary file 1 — Supplementary Material 1. [file 12885_2025_15342_MOESM1_ESM.docx]

**PATIENT GUIDE**

**Issue to be discussed:** Implementation of a physical activity program during treatment for breast cancer.

1. **INTRODUCTION**

Thank you for taking the time to be interviewed for the IMPACT Breast Cancer Study today. My name is [NAME] and I will lead you through the questions. [Interviewer to briefly introduce self & “meet” participant].

**Purpose**

We want to know more about the benefits, drawbacks, challenges, and important supporters of delivering a physical activity program to patients with breast cancer who are getting chemotherapy. We hope to learn more about the type of program to build and how to deliver it in the best way. Our long-term goal is to develop a lifestyle program that can be easily delivered to patients as a standard part of their treatment plan. We truly value your input and it will help us understand and accommodate for issues in future physical activity programs.

**Informed Consent Information**

This is a safe environment, and we will not tell anyone what specific people say here. We will use the information you give us to write a scientific paper, but your names will not be included nor will any identifying information about you. You do not have to answer any questions that you do not want to answer. You can skip the questions you do not want to answer and still stay in this discussion group. If you do not want to participate, you do not have to. What questions do you have so far?

**Notetaking & Recording**

Since we are writing a report from these talks, I will also record this session so I don’t miss anything. [Start recording].

*Ask if participant has any further questions – if not, begin the interview*

1. **GENERAL QUESTIONS ABOUT THE TOPIC**

These questions are designed to help us understand physical activity interventions and set the context for our discussion.

*[Primarily taken from Characteristics of Individuals construct from CFIR].*

**Knowledge and beliefs about the intervention**

- What does a physically active lifestyle look like to you?
  - Probe: How does being physically active impact your health?
  - Probe: How does *not* being physically active impact your health?
- How do you feel about physical activity being a part of treatment for breast cancer?
- Do you think physical activity will be effective at improving your health during chemotherapy?
  - Probe: What could your healthcare provider do to help you be active?

**Self-efficacy**

- How confident are you that you could be part of a physical activity program while getting chemotherapy?
  - Follow-up: What would you say is the biggest factor impacting your confidence?
- How confident would you *have been* that you could be part of a physical activity program before you got your diagnosis?

**Individual stage of change**

- What are some things that would make you feel ready to participate in a physical activity program during chemotherapy?
- What are some things that would make you feel *not ready* to participate in a physical activity program during chemotherapy?

1. **QUESTIONS ABOUT INTERVENTION CHARACTERISTICS**

**General**

- What motivates you to be active?
- What features do you think will be important to include in a program that aims to increase physical activity during chemotherapy?
- Who would you prefer deliver this type of physical activity program? (Ex: doctor or nurse practitioner, physical therapist, community center)
- If you were going to join a physical activity program while getting chemotherapy, would you prefer an individual (one-on-one) program or a group program?

**Evidence strength & quality**

- What kind of information are you aware of that shows whether or not physical activity will work for improving your health during chemotherapy?
- What kind of evidence for physical activity would you like to see to get you on board?
  - Probe: This could be support from your doctor, family and friends, scientific articles

**Design quality & packaging**

- What supports, such as peer support, support from your care team, or online resources, would you need to be successful at completing a physical activity program during chemotherapy?

1. **QUESTIONS ABOUT OUTER SETTING**

**Patient needs & resources**

- How well do you think a physical activity intervention during chemotherapy will meet the needs of other patients?
- What barriers will patients face to participating in this sort of intervention?
  - Probe: Would in-person vs. remote delivery affect barriers?
- What facilitators will patients face to participating in this sort of intervention?
- Have you heard stories about the experiences of patients who were physically active during chemotherapy?

1. **QUESTIONS ABOUT PROCESS**

- What recommendations do you have to successfully enroll patients during chemotherapy?
- How would you like to hear about this intervention?
- What would make it easy for you to sign-up for this intervention?
- Do you think it’s reasonable to deliver an intervention during this time?
  - Why/why not?
- What are important considerations for research teams to know about delivering programs during chemotherapy?
- Did we miss anything that you would like to share with us today?

1. **THANK YOU AND CLOSE**

Thank you for your time today. I have learned a lot from you, and I know what you shared today will help us design an effective physical activity program for patients diagnosed with breast cancer. [End recording].

**CLINICIAN GUIDE**

**Issue to be discussed:** **Implementation of a physical activity program during treatment for breast cancer.**

1. **INTRODUCTION**

Thank you for taking the time to be interviewed for the IMPACT Breast Cancer Study today. My name is [NAME] and I will lead you through the questions. [Interviewer to briefly introduce self & “meet” participant].

**Purpose**

We want to know more about the benefits, drawbacks, challenges, and important supporters of delivering a physical activity program to patients with breast cancer who are getting chemotherapy. We hope to learn more about the type of program to build and how to deliver it in the best way. Our long-term goal is to develop a lifestyle program that can be easily delivered to patients as a standard part of their treatment plan. We truly value your input and it will help us understand and accommodate for issues in future physical activity programs.

**Informed Consent Information**

This is a safe environment, and we will not tell anyone what specific people say here. We will use the information you give us to write a scientific paper, but your names will not be included nor will any identifying information about you. You do not have to answer any questions that you do not want to answer. You can skip the questions you do not want to answer and still stay in this discussion group. If you do not want to participate, you do not have to. What questions do you have so far?

**Notetaking & Recording**

Since we are writing a report from these talks, I will also record this session so I don’t miss anything. [Start recording].

**Maintain neutrality. Probe if they mention a specific program, referral practice, etc.*

**I am now starting the recording. This is health care stakeholder interview #X.**

**Before we begin, can you please tell me your official title and provide a brief overview of your role? At which clinic sites do you practice? What is your interest in improving survivorship care?**

1. **GENERAL QUESTIONS ABOUT THE TOPIC**

**In this first section, we’ll just be covering some general questions that are designed to help us further describe what we are interested in understanding about physical activity interventions and set the context for the rest of our discussion.**

**Knowledge and beliefs about the intervention**

- **So to begin, what does a physically active lifestyle look like to you?** ****Look for health impact***
  - ***Probe:* How does being physically active impact your health?**
  - ***Probe:* How does *not* being physically active impact your health?**
- **How do you feel about physical activity being a part of standard treatment for breast cancer?**
  - ***Probe* – *If not sure how they feel:* Do you think physical activity will be effective at improving patients’ health during chemotherapy?**
  - ***Probe:* Why/why not?**
  - ***Probe:* What do you know about other standard of care physical activity programs, such as cardiac rehab?**

**Self-efficacy**

- **How confident are you that you would be able to successfully refer patients to, or implement, a physical activity program during chemotherapy? **Look for biggest factor impacting referral confidence***
  - ***Follow-up if they are confident:* What would you say is the biggest factor impacting your confidence?**
- ***Follow-up if they are NOT confident:* What are some things that would make you feel ready to refer patients to, or implement, a physical activity program during chemotherapy?**

1. **QUESTIONS ABOUT INTERVENTION CHARACTERISTICS**

**Next, I am going ask some questions about the characteristics of an intervention.**

**General**

- **What motivates you to refer patients to physical activity programs during chemotherapy?**
- **Who would you prefer deliver this type of physical activity program? (Ex: doctor or nurse practitioner, physical therapist, community center)**
  - ***Probe:* Why? Is it due to patient buy-in, feasibility of referrals, patient safety?**
  - ***Probe - if they did not already mention patient safety:* Are you concerned about patients’ safety completing a physical activity program during chemotherapy?**
- **IF APPROPRIATE: What kind of features do you think will be important to include in a program that aims to increase physical activity during chemotherapy?**
  - ***Probe:* Do you prefer individual or group programs?**

**Relative advantage**

- **Have you heard about other lifestyle programs that patients could be referred to?**
  - ***Probe - If yes:***
    - **What do you like about it? Are there certain things that worked well? What do you not like about it?**
    - **Did patients like it/why? Are there things that they didn’t like about it?**

**Evidence strength & quality**

- **What kind of information are you aware of that shows whether or not physical activity will work for improving patients’ health during chemotherapy?**
  - **If not aware of this type of information: What kind of evidence for physical activity would you like to see to get you on board?**
    - ***Probe:* This could be support/buy-in from your colleagues, scientific articles**
    - ***Probe:* How would you like to receive this sort of information? Training, conference, email info, etc.?**

**Cost**

- **What costs would be incurred to implement such an intervention?**
  - ***Probe:* Would these costs be prohibitive?**
    - ***Probe:* Are there other indirect costs, such as time away from family, travel, time off work?**
- ***Probe:* Do you worry about insurance reimbursement when referring patients to lifestyle programs during chemotherapy?**

1. **QUESTIONS ABOUT OUTER SETTING**

**Now I have just a few questions about patient needs and resources.**

**Patient needs & resources**

- **How well do you think this sort of intervention will meet the needs of patients?**
- **What barriers will patients face to participating in this sort of intervention?**
  - ***Probe:* Would in-person vs. remote delivery affect barriers?**
- **What facilitators will help patients participate in this sort of intervention?**
- **Have you heard stories about the experiences of patients who were physically active during chemotherapy?**

1. **QUESTIONS ABOUT INNER SETTING**

**Shifting to the internal setting, we’re going to talk a bit about culture and implementation readiness.**

**Culture**

- **How do you think your healthcare system’s beliefs, values and assumptions about physical activity will affect implementation during chemotherapy?**

**Readiness for implementation**

- **Do you expect to have sufficient resources in your department/division to implement a physical activity program during chemotherapy?**
  - **Probe: What about in a non-COVID scenario?**
- **Who do you ask if you have questions about referring patients to physical activity?**

1. **QUESTIONS ABOUT PROCESS**

**And finally, I have just a few process-related questions for you.**

- **What recommendations do you have to successfully enroll patients in a physical activity during chemotherapy?**
- **What would make it easy for you to refer patients to a physical activity program during chemotherapy?**
- **Do you think it’s reasonable to deliver an intervention during chemotherapy?**
  - ***Probe:* Why/why not?**
- **What are important considerations for research teams to know about delivering programs during chemotherapy?**
- **Did we miss anything that you would like to share with us today?**

1. **THANK YOU AND CLOSE**

That concludes our interview. I want to again thank you for your time today.

I have learned a lot from you, and I know what you shared today will help us design an effective physical activity program for patients diagnosed with breast cancer.

***I am now ending the recording. [End recording].***
